# Comparison of Immediate and Sequential Withdrawal of a Systemic Glucocorticoid in the Treatment of Acute Exacerbations of Chronic Obstructive Pulmonary Disease: A Multicenter, Randomized, Double-Blind, Parallel-Controlled, Open-Label Study

**DOI:** 10.3389/fmolb.2021.639079

**Published:** 2021-05-20

**Authors:** Ling Zhou, Yuanyuan Fang, Wei Liu, Jianchu Zhang, Yingnan Wang, Sheng Xie, Minhua Zhong, Zhengyan Wang, Guangcai Li, Hongyan Ai, Hongrong Guo, Fanjun Zeng, Wei Xiao, Chenghong Li, Yi Hu, Yijun Tang, Huiguo Liu

**Affiliations:** ^1^Department of Respiratory and Critical Care Medicine, Tongji Hospital, Tongji Medical College, Huazhong University of Science and Technology, Wuhan, China; ^2^Department of Respiratory and Critical Care Medicine, Renmin Hospital of Wuhan University, Wuhan, China; ^3^Department of Respiratory and Critical Care Medicine, Union Hospital, Tongji Medical College, Huazhong University of Science and Technology, Wuhan, China; ^4^Department of Respiratory and Critical Care Medicine, The People’s Hospital of China Three Gorges University, The First People’s Hospital of Yichang, Yichang, China; ^5^Department of Respiratory and Critical Care Medicine, Huangshi Central Hospital, Affiliated Hospital of Hubei Polytechnic University, Huangshi, China; ^6^Department of Respiratory and Critical Care Medicine, Xiaogan Hospital Affiliated to Wuhan University of Science and Technology, The Central Hospital of Xiaogan, Xiaogan, China; ^7^Department of Respiratory Medicine, Suizhou Hospital, Hubei University of Medicine, Suizhou, China; ^8^Department of Respiratory and Critical Care Medicine, The Central Hospital of Enshi Tujia and Miao Autonmous Prefecture, Enshi Clinical College of Wuhan University, Enshi Tujia and Miao Autonomous Prefecture, Hubei, China; ^9^Department of Respiratory and Critical Care Medicine, Hanyang Hospital Affiliated to Wuhan University of Science and Technology, Wuhan, China; ^10^Department of Respiratory and Critical Care Medicine, Wuhan Third Hospital, Tongren Hospital of Wuhan University, Wuhan, China; ^11^Department of Respiratory and Critical Care Medicine, The First College of Clinical Medicine Science, China Three Gorges University, Yichang Central People’s Hospital, Yichang, China; ^12^Department of Respiratory and Critical Care Medicine, The First People’s Hospital of Jingzhou, Jingzhou, China; ^13^Department of Respiratory and Critical Care Medicine, The Sixth Hospital of Wuhan, Jianghan University, Wuhan, China; ^14^Department of Respiratory and Critical Care Medicine, The Central Hospital of Wuhan, Tongji Medical College, Huazhong University of Science and Technology, Wuhan, China; ^15^Department of Respiratory and Critical Medical, Taihe Hospital, Affiliated Hospital of Hubei University of Medicine, Shiyan, China

**Keywords:** glucocorticoids, immediately withdrawal, sequential withdrawal, acute exacerbations of chronic obstructive pulmonary disease, prognosis, methyl predrone, Central China

## Abstract

Patients with acute exacerbations of chronic obstructive pulmonary disease (AECOPD) were treated with immediate or sequential withdrawal after 5 days of systemic glucocorticoids. The effects of the two withdrawal methods on the prognosis of patients were compared at 30, 90, 180, and 360 days after discharge. A multicenter, randomized, double-blind, parallel-controlled, open-label study was conducted in the respiratory department of tertiary hospitals in Central China. Patients met inclusion criteria for AECOPD and needed to use systemic glucocorticoids. They were randomly assigned to immediate and sequential withdrawal groups at a 1:1 ratio. The study was completed in August 2020 and is registered at the China Clinical Trials Registry (Chictr.org) (ChiCTR1800018894). According to general data and clinical characteristics, there were no statistically significant differences between the 329 patients in the immediate withdrawal group and the 310 patients in the sequential withdrawal group (*P* > 0.05). At the 30, 90, 180, and 360-days follow-up, the acute exacerbation frequency, rehospitalization rate, mortality, and intensive care unit (ICU) treatment rate were not significantly different between the immediate withdrawal group and sequential withdrawal group (*P* > 0.05). The modified Medical Research Council (mMRC) and COPD assessment test (CAT) scores were also not significantly different between the two groups. At the 180- and 360-day follow-up, forced expiratory volume in 1 s (FEV1%) and peak expiratory flow (PEF) were not significantly different between the two groups (*P* > 0.05). The time from discharge to first acute exacerbation was significantly lower in the immediate withdrawal group (46.12 days) than in sequential withdrawal group (49.02 days) (*P* < 0.05). The time of stay in the hospital for the first time after discharge was not significantly different between the two groups (*P* > 0.05). Adverse events were not significantly different between the immediate withdrawal group and sequential withdrawal group (*P* < 0.05). Subgroup analysis was performed according to age, degree of disease, and relevant indicators. At the 30-day follow-up, the acute exacerbation frequency of patients with advanced age, high global strategy for chronic obstructive lung disease (GOLD), and high fractional exhaled nitric oxide was significantly higher in the immediate withdrawal group than in the sequential withdrawal group (*P* < 0.05). In addition, according to receiver operating characteristic (ROC) curve analysis, the frequency of acute exacerbations at the 30-day follow-up was significantly higher in patients with age > 63.5 years or GOLD > 3 in the immediate withdrawal group than in the sequential withdrawal group, suggesting that the short-term efficacy was poor.

## Introduction

Chronic obstructive pulmonary disease (COPD) is a chronic, non-specific disease in which inflammation of the airways activates inflammatory epithelial and smooth muscle cells and the release of inflammatory mediators (Doumas et al., 2020). Considering its high rates of morbidity and disability, COPD can pose a serious threat to human health (Rabe et al., 2020). Acute exacerbations of chronic obstructive pulmonary disease (AECOPD) occur when COPD patients show rapid changes within a short period (Vermeersch et al., 2019), with obvious aggravation of clinical symptoms such as cough, sputum production, dyspnea, and shortness of breath. In the absence of timely treatment, the life of the patient may be in jeopardy (Berenyi et al., 2020).

The Global Strategy for Chronic Obstructive Lung Disease (GOLD) guide indicates that glucocorticoids can shorten recovery time and improve lung function and hypoxia during acute exacerbations, in addition to reducing the risk of recurrence and disease progression (Burns et al., 2020). However, the long-term use of glucocorticoids causes a variety of adverse events, such as hyperglycemia, necrosis of the femoral head, reduction in immunity, and increased risk of infection (Lucafo et al., 2020). The GOLD guide recommends methyl prednisone at 40 mg/d for 5 days in cases of AECOPD (Leuppi et al., 2013). The United Kingdom National Institute for Health and Care Excellence (NICE) recommended that COPD be treated with systemic glucocorticoids for 7–14 days (National Collaborating Centre for Chronic, 2004). The Australian and New Zealand guidelines for the diagnosis and treatment of COPD recommend an optimal course of systemic glucocorticoids for 10–14 days (McKenzie et al., 2003). Thus, there is still no consensus on the treatment time and dose of systemic glucocorticoids in the treatment of AECOPD. In this study, a randomized controlled trial was used to compare the short-term efficacy and long-term prognosis of immediate and sequential withdrawal of methyl prednisone treatment of AECOPD.

## Materials and Methods

### Study Design and Subjects

A multicenter, randomized, double-blind, parallel-controlled study was conducted in tertiary hospitals in Central China to compare the effects of systemic glucocorticoid immediate or sequential withdrawal on the prognosis of AECOPD patients at 30, 90, 180, and 360 days after discharge. Patients admitted from July 2017 to August 2019 were randomly assigned to the immediate and sequential withdrawal groups at a 1:1 ratio. Patients in the immediate withdrawal group received intravenous injections of methyl predrone at 40 mg/d for five consecutive days, then withdrawal on day six. Patients in the sequential withdrawal group received the same 5-day treatment, but the dose was reduced to 30 mg/d on days six and seven, to 20 mg/d on days eight and nine, and then stopped completely on day 10. The immediate group received the same amount of saline injection as the sequential withdrawal group from 6 to 10 days. To evaluate the efficacy and prognosis of the two treatment regiments, patient follow-ups were conducted for 1 year from the discharge date. The competent authorities of participating hospitals and the Medical Ethics Committee of Tongji Medical College of Huazhong University of Science and Technology approved the study (2018-S301). The study is registered at the China Clinical Trials Registry (Chictr.org) (ChiCTR1800018894).

All patients agreed to participate in the study and signed the informed consent. The inclusion criteria were as follows: (1) age between 40 years and 70 years; (2) diagnosis of AECOPD: sudden changes in clinical symptoms, including dyspnea, cough, and expectoration of sputum; (3) COPD beyond the range of daily variation; (4) pulmonary function examination indicating that forced expiratory volume in 1 s (FEV1%) < 0.70 after previous use of a bronchodilator. Patients suffering from the following diseases or lesions were excluded: (1) those critically ill patients with assisted respiratory muscle involvement in respiratory movement, thoracic and abdominal contradictory breathing, cyanosis, or need for invasive mechanical ventilation; (2) those with edema, right heart failure, or hemodynamic instability; (3) those with changes in mental state; (4) those with malignant tumors or other serious diseases.

### Randomization, Double Blindness, and Follow-ups

The study leader LHG placed the subjects in order of inclusion in either the sequential withdrawal or the immediate withdrawal group. The study leader did not participate in subject recruitment and kept the randomization form confidential. Since the subjects signed the consent form, the researchers and the subjects were informed of the grouping.

Baseline measurements were taken on the day of recruitment, and clinical symptoms were assessed daily during hospitalization (cough assessment score, sputum viscosity, sputum volume, mMRC score). Discharge decisions were made by the attending physician. Clinical symptoms, mMRC score, and CAT score were assessed on days 30, 90, 180, and 360 after discharge, with the frequency of acute exacerbations from the last follow-up recorded. Pulmonary function (FEV1%,PEF) was measured on days 180 and 360 after discharge. There was a focus on adverse events (hypertension, kidney injury, electrolyte disturbance, infection, edema, plasma glucose, and osteoporosis) during and after therapy.

### Endpoints

The primary endpoint was the frequency of acute exacerbations at 30, 90, 180, and 360 days after discharge. The key secondary endpoint was mortality. Other secondary endpoints were the mMRC score, CAT score, rehospitalization rate, and ICU treatment rate at 30, 90, 180, and 360 days after discharge and FEV1% and PEF at 180 and 360 days after discharge.

### Statistical Analyses

Categorical variables were presented as numbers (percentages) and analyzed using a chi-squared test or Fisher’s exact test. Continuous variables with a normal distribution were expressed as the mean ± standard deviation and analyzed using the independent samples *t*-test, whereas those with a skewed distribution were shown as the median (Q_1_, Q_3_) and analyzed using the Mann–Whitney U test. COX regression analyses were adopted to identify risk factors of prognosis. Receiver operating characteristic (ROC) curves were used to calculate the area under the curve (AUC) of a single continuous variable and the cutoff value. The SPSS software v 25.0 (SPSS Inc., Chicago, IL, United States) was used for statistical analyses. Differences at *P* < 0.05 (two-sided) were considered statistically significant.

## Results

### General Data and Clinical Characteristics

**FIGURE 1:**
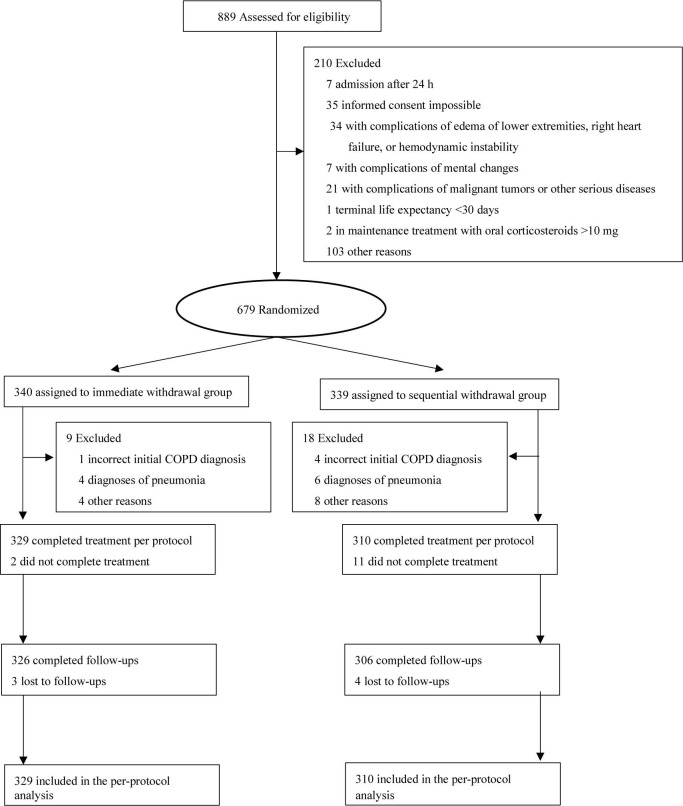
Trial participant enrollment, assignment to immediate and sequential withdrawal groups, and inclusion in follow-ups.

Trial participant assignment to immediate and sequential withdrawal groups, and inclusion in follow-ups are shown in Figure 1. There were 639 COPD patients, including 349 males and 290 females. The average age was 61.02 years, and the average body mass index was 26.03. There were 230 patients that had smoked in the past and 62 patients that were smoking at the time of the study, with 14.93 years being the average time of smoking. Forty-nine cases had COPD exacerbations more than three times in the previous year. Fifteen cases had a history of exposure to biofuels. Among these indicators, there were no statistically significant differences between the immediate and sequential withdrawal group (*P* > 0.05). In addition, the Charlson comorbidity index, admission characteristics, pulmonary function indicators, GOLD grade, mMRC score, CAT score, laboratory indicators, and use of home oxygen therapy were not significantly different between the two groups (*P* > 0.05). We focus on hypertension, kidney injury, electrolyte disturbance, infection, edema, plasma glucose, and osteoporosis in this study, but there were no statistically significant differences between the immediate withdrawal group and sequential withdrawal group (*P* > 0.05, Table 1).

**TABLE 1:** Baseline characteristics of the intention-to-treat population.

Items	Total (*n* = 639)	Immediate withdrawal (*n* = 329)	Sequential withdrawal (*n* = 310)	Statistic	*P*
Age (y)	61.02 ± 0.19	61.19 ± 0.28	60.87 ± 0.27	0.802*	0.423
**Sex**					
Male	349 (54.6)	177 (53.8)	172 (55.5)	0.183^†^	0.669
Female	290 (45.4)	152 (46.2)	138 (44.5)		
BMI	26.30 ± 0.18	26.00 ± 0.26	26.61 ± 0.27	1.597*	0.111
**Smokers**					
Never	347 (54.3)	178 (54.1)	169 (54.5)	0.779^†^	0.677
Past	230 (36.0)	122 (37.1)	108 (34.8)		
Current	62 (9.7)	29 (8.8)	33 (10.7)		
Pack-years smoked (y)	14.93 ± 0.48	14.85 ± 0.70	15.01 ± 0.65	0.167*	0.868
Course of COPD (y)	3.38 ± 0.06	3.38 ± 0.08	3.36 ± 0.09	0.217*	0.829
Number of AECOPD in previous year				2.837^†^	0.586
0	45 (7.0)	24 (7.3)	21 (6.8)		
1	74 (11.6)	41 (12.5)	33 (10.6)		
2	292 (45.7)	156 (47.4)	136 (43.9)		
3	179 (28.0)	83 (25.2)	96 (31.0)		
> 3	49 (7.7)	25 (7.6)	24 (7.7)		
History of biofuel use	15 (2.3)	6 (1.8)	9 (2.9)	0.811^†^	0.368
Charlson comorbidity index	2.86 ± 0.03	2.87 ± 0.04	2.86 ± 0.05	0.834*	0.834
**Admission presentation**					
Cough symptom score	2.05 ± 0.04	2.09 ± 0.05	2.01 ± 0.04	1.207*	0.228
Sputum viscosity	1.98 ± 0.03	2.02 ± 0.06	1.92 ± 0.05	1.393*	0.164
Sputum volume	1.66 ± 0.02	1.62 ± 0.03	1.70 ± 0.04	1.195*	0.232
Pulmonary function					
FEV1%	60.34 ± 0.27	59.99 ± 0.40	60.71 ± 0.35	1.360*	0.174
PEF	7.76 ± 0.08	7.62 ± 0.11	7.91 ± 0.10	1.852*	0.065
GOLD				7.273^†^	0.064
GOLD1	30 (4.6)	14 (4.3)	16 (5.2)		
GOLD2	229 (35.9)	105 (31.9)	124 (40.0)		
GOLD3	275 (43.0)	158 (48.0)	117 (37.7)		
GOLD4	105 (16.5)	52 (15.8)	53 (17.1)		
mMRC score	1.99 ± 0.03	1.96 ± 0.04	2.03 ± 0.05	0.997*	0.319
CAT score	18.21 ± 0.29	18.11 ± 1.15	17.18 ± 0.46	0.814*	0.416
**Laboratory**					
Leucocytes (10^9^/L)	6.83 ± 0.11	6.66 ± 0.16	7.02 ± 0.14	1.687*	0.092
Eosinophils (10^9^/L)	0.55 ± 0.05	0.55 ± 0.03	0.56 ± 0.11	0.129*	0.897
C-reactive protein (mg/L)	2.50 ± 0.16	2.22 ± 0.18	2.69 ± 0.24	1.475*	0.141
FeNO	29.62 ± 0.71	31.05 ± 1.08	28.10 ± 0.91	0.454*	0.224
PaCO_2_ (kPa)	40.32 ± 0.31	39.91 ± 0.401	40.92 ± 0.50	1.589*	0.114
PaO_2_ (kPa)	66.82 ± 1.35	67.11 ± 1.95	66.38 ± 1.73	0.265*	0.791
Home oxygen therapy	76 (11.9)	34 (10.3)	42 (13.5)	1.573*	0.209
**Clinical findings**					
Diastolic blood pressure (mm Hg)	79 ± 0.36	78 ± 0.56	80 ± 0.44	1.273*	0.204
Systolic blood pressure (mm Hg)	116 ± 0.32	116 ± 0.42	117 ± 0.49	1.168*	0.243
Heart rate (beats/min)	92 ± 0.47	91 ± 0.67	92 ± 0.46	0.659*	0.510
Oxygen saturation (%)	94 ± 0.24	94 ± 0.39	95 ± 0.27	1.931*	0.054
Respiratory rate (breaths/min)	21 ± 0.71	21 ± 0.34	20 ± 0.35	0.863*	0.389
**Adverse events**					
Hypertension	0	0	0	–	–
Electrolyte disturbance	3 (0.5)	2 (0.6)	1 (0.3)	0.278^†^	0.598
Kidney injury	0	0	0	–	–
Infection	0	0	0	–	–
Edema	4 (0.6)	1 (0.3)	3 (1.0)	1.131^†^	0.287
Elevated plasma glucose	6 (0.9)	2 (0.6)	4 (1.3)	0.667^†^	0.414
Osteoporosis	0	0	0	–	–

Data are shown as the mean ± standard deviation or n (%). *t-test; ^†^χ^2^-value. BMI, Body Mass Index; COPD, chronic obstructive pulmonary disease; AECOPD, acute exacerbation of chronic obstructive pulmonary disease; FEV1, forced expiratory volume in 1 s; PEF, peak expiratory flow; GOLD, global initiative for chronic obstructive lung disease;mMRC, Modified Medical Research Council Dyspnea Scale; CAT, COPD Assessment Test; FeNO, Fractional exhaled nitric oxide; PaO_2_, partial pressure of oxygen; PaCO_2_, partial pressure of carbon dioxide. Degree of sputum viscosity: (1), sputum clings to the wall of phlegm cup during rotation; (2), sputum slides slowly along the wall of phlegm cup during rotation; (3), sputum can be poured out quickly but some remains attached to the wall of phlegm cup; (4), sputum can be poured out quickly and completely. Sputum volume: 1, mild, with less than 10 mL/day; 2, moderate, with 10–150 mL/day; 3, severe, with more than 150 mL/day.

### Endpoints

**FIGURE 2:**
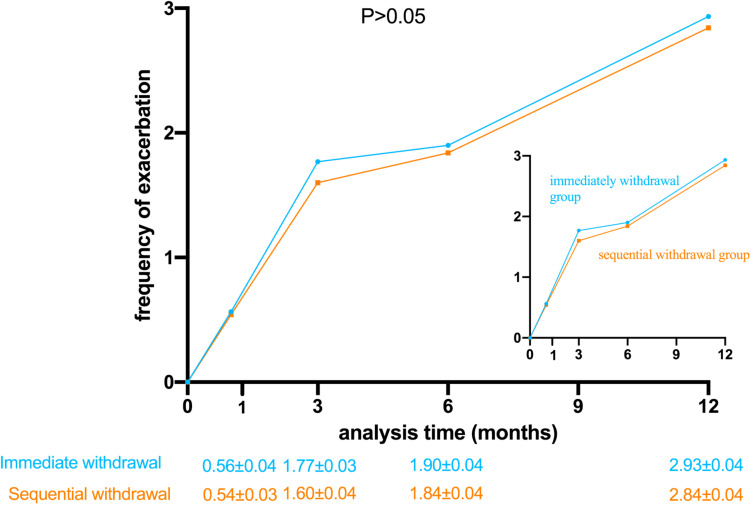
Frequency of acute exacerbations in the immediate withdrawal group and the sequential withdrawal group with time following discharge. (*P* > 0.05).

The primary endpoint was the frequency of acute exacerbations in the follow-ups at 30, 90, 180, and 360 days after discharge. The frequency of acute exacerbations was not significantly different between immediate and sequential withdrawal groups at 30, 90, 180, and 360 days after discharge (*P* > 0.05, Figure 2).

The key secondary endpoint was mortality. Mortality was not significantly different between immediate and sequential withdrawal groups at 30, 90, 180, and 360 days (*P* > 0.05). The other secondary endpoints were mMRC score, CAT score, admission rate, and ICU treatment rate at 30, 90, 180, and 360 days after discharge and FEV1% and PEF at 180 and 360 days after discharge. There were no statistically significant differences in these indicators between the two groups (*P* > 0.05). The time from discharge to first acute exacerbation was significantly lower in the immediate withdrawal group (46.12 days) than in the sequential withdrawal group (49.02 days) (*P* < 0.05). The time of stay in the hospital for the first time after discharge was longer in the immediate withdrawal group (6.28 days) than in the sequential withdrawal group (6.17 days), but the difference was not statistically significant (*P* > 0.05, Table 2).

**TABLE 2:** Secondary endpoints.

	Visit time (days)	Immediate withdrawal group	Sequential withdrawal group	Statistic	*P*-value
**Key secondary endpoint**					
Mortality	30	0	0	–	–
	90	3 (0.9)	3 (1)	0.073*	0.942
	180	5 (1.5)	6 (1.9)	1.163^†^	0.686
	360	7 (2.1)	8 (2.6)	0.143^†^	0.705
**Other secondary endpoints**					
mMRC score	30	1.97 ± 0.05	1.99 ± 0.05	0.380^‡^	0.704
	90	2.01 ± 0.05	2.06 ± 0.05	0.679^‡^	0.497
	180	1.95 ± 0.04	2.08 ± 0.05	1.923^‡^	0.055
	360	1.96 ± 0.05	2.04 ± 0.05	0.997^‡^	0.319
CAT score	30	18.93 ± 0.38	17.15 ± 0.44	3.045^‡^	0.770
	90	19.31 ± 0.37	17.61 ± 0.45	2.875^‡^	0.841
	180	19.04 ± 0.36	17.35 ± 0.47	2.854^‡^	0.312
	360	19.09 ± 0.36	17.66 ± 0.49	2.354^‡^	0.162
Rehospitalization rate	30	63 (19.1)	44 (14.2)	2.811^†^	0.094
	90	107 (32.5)	90 (29.0)	0.912^†^	0.339
	180	123 (37.4)	132 (42.6)	1.796^†^	0.180
	360	144 (43.8)	145 (46.8)	0.582^†^	0.446
ICU treatment rate	30	3 (0.9)	6 (1.9)	1.097*	0.328
	90	12 (3.6)	15 (4.8)	0.559^†^	0.454
	180	25 (7.6)	26 (8.4)	0.135^†^	0.713
	360	47 (14.3)	45 (14.5)	0.007^†^	0.934
FEV1%	180	61.00 ± 0.48	61.80 ± 0.42	1.501^‡^	0.134
	360	62.92 ± 0.51	61.57 ± 0.47	1.665^‡^	0.097
PEF	180	7.54 ± 0.11	7.89 ± 0.14	1.263^‡^	0.207
	360	7.73 ± 0.15	7.89 ± 0.14	1.075^‡^	0.283
Time from discharge to first acute exacerbation	–	46.12 ± 1.28	49.02 ± 1.33	0.489^‡^	0.030
Time of stay in hospital for the first time after discharge	–	6.28 ± 0.07	6.17 ± 0.05	1.275^‡^	0.203

Data are shown as the mean ± standard deviation or n (%). *Fisher’s test; ^†^χ^2^-value; ^‡^t-test. mMRC, Modified Medical Research Council Dyspnea Scale; CAT, COPD Assessment Test; ICU, intensive-care unit; FEV1, forced expiratory volume in 1 s; PEF, peak expiratory flow.

### Subgroup Analysis

#### Subgroup Analysis at the 30-day Follow-Up

**FIGURE 3:**
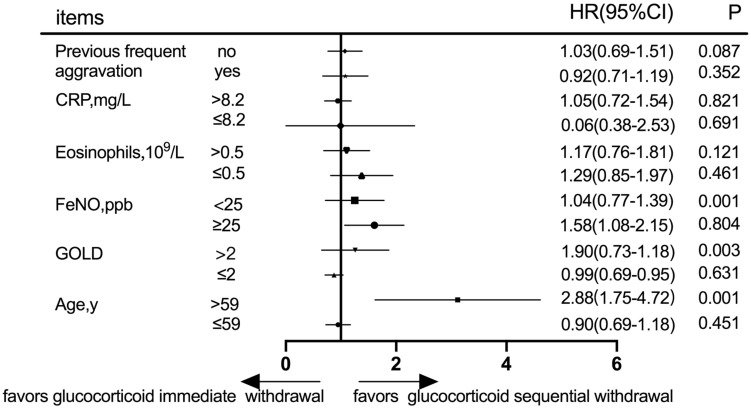
Subgroup analysis according to previous frequent aggravation, CRP, eosinophils, FeNO, GOLD grade, and age at the 30-day follow-up. CRP, C-reactive protein; FeNO, Fractional exhaled nitric oxide; GOLD, global initiative for chronic obstructive lung disease.

Prognostic evaluation was defined according to acute exacerbation frequency in the follow-up at 30 days after discharge. Subgroup analysis was performed according to the history of frequent acute exacerbations, C-reactive protein (CRP), eosinophils, fractional exhaled nitric oxide (FeNO), GOLD grade, and age (Figure 3). In patients with either frequent or infrequent acute exacerbation history, high CRP or low CRP, high eosinophils or low eosinophils, there was no statistically significant difference in acute exacerbation frequency between immediate and sequential withdrawal groups at the 30-day follow-up (*P* > 0.05). Although the acute exacerbation frequency of patients with high FeNO showed no statistically significant difference between the two groups (*P* > 0.05), in patients with low FeNO, the difference was significant between immediate and sequential withdrawal groups (*P* < 0.05). Although the acute exacerbation frequency of patients with a low GOLD grade was not different between the two withdrawal groups, in patients with a high GOLD grade, the difference was significant between immediate and sequential withdrawal groups at the 30-day follow-up (*P* < 0.05). The acute exacerbation frequency in patients with age ≤ 59 years showed no statistically significant difference between the immediate and sequential withdrawal groups (*P* > 0.05). However, in patients with age > 59 years, the difference between withdrawal methods was significant (*P* < 0.05). Thus, the results of the 30-day follow-up show acute exacerbation frequency in patients with advanced age, high GOLD grade, and low FeNO, and was significantly higher in the immediate withdrawal group than in the sequential withdrawal group (*P* < 0.05, Figure 3).

#### Subgroup Analysis at the 360-day Follow-up

**FIGURE 4:**
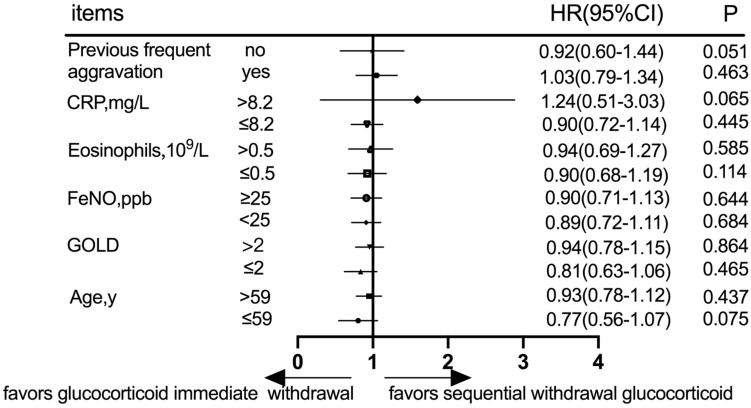
Subgroup analysis according to previous frequent aggravation, CRP, eosinophils, FeNO, GOLD grade, and age at the 360-day follow-up. CRP, C-reactive protein; FeNO, Fractional exhaled nitric oxide; GOLD, global initiative for chronic obstructive lung disease.

Prognostic evaluation was defined according to acute exacerbation frequency in the follow-up at 360 days. Subgroup analysis was performed according to the history of frequent acute exacerbations, CRP, eosinophils, FeNO, GOLD grade, and age (Figure 4). In patients with a history of either previous frequent aggravation or infrequent aggravation, there was no statistically significant difference in acute exacerbation frequency between immediate and sequential withdrawal groups at the 360-day follow-up (*P* > 0.05). Similarly, in patients with either high or low CRP, high or low eosinophils, high or low FeNO, high or low GOLD grade, age ≤ 59 years or age > 59 years, there was no statistically significant difference in acute exacerbation frequency between immediate and sequential withdrawal groups at the 360-day follow-up (*P* > 0.05, Figure 4).

### Efficacy Analysis of Each Indicator at the 30-day Follow-up

**FIGURE 5:**
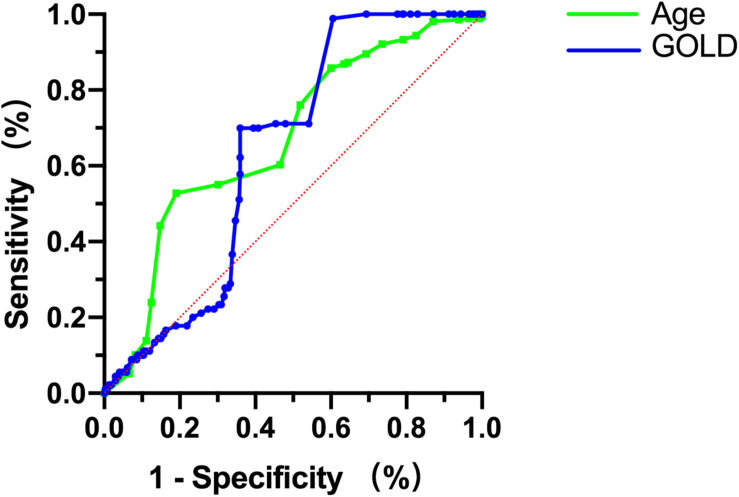
Receiver operating characteristic curves for age and GOLD in the diagnosis of short-term efficacy.

Subgroup analysis showed that different ages, GOLD grading, and FeNO values could affect short-term efficacy. ROC curve analysis was performed based on the significant index values (age, GOLD, FeNO) to analyze short-term efficacy. Although FeNO had no diagnostic value for short-term efficacy (*P* > 0.05), age and GOLD grading had diagnostic value in evaluating the short-term efficacy (*P* < 0.05). Thus, age > 63.5 years with an AUC = 0.668 (95%CI: 0.627–0.711) and GOLD > 3 with an AUC = 0.638 (95%CI: 0.586–0.689) were risk factors for short-term efficacy (Figure 5 and Table 3).

**TABLE 3:** Efficacy analysis of each indicator at the 30-day follow-up.

Items	AUC (95%CI)	*P*-value	Cut-off	Sensitivity (%)	Specificity (%)	Yoden index
Age	0.668 (0.627–0.711)	0.001	63.5	52.81	81.96	0.347
GOLD	0.638 (0.586–0.689)	0.007	3	40.47	98.89	0.393
FeNO	0.541 (0.497–1.586)	0.067	/	/	/	/

## Discussion

The treatment goal for AECOPD is to minimize the adverse effects of the current deterioration of the disease as soon as possible and prevent the development of subsequent events (Zhang et al., 2020). Standardized diagnosis and treatment measures play an important role in the prognosis of patients. Current recommendations from the Global Initiative for Chronic Obstructive Lung Disease suggest that patients who continue to have exacerbations should initially step up to glucocorticoid therapy (Terry and Dhand, 2019, 2020). GOLD point out AECOPD treatment strategies: “A dose of 40 mg prednisone per day for 5 days is recommended.” and “Intensified combination therapy with ICS/LABA for 10 days at Upper Respiratory Tract Infection onset could be associated with a reduction of exacerbations, particularly in patients with severe disease” (Singh et al., 2019). Immediate withdrawal for 5-days can reduce the treatment dose of glucocorticoid, but rapid withdrawal or dose reduction may result in a rebound effect, causing aggravation and deterioration of the original disease. Prolonged use of glucocorticoid can reverse severe illness and reduce the acute acerbation frequency in specific subgroups. some diseases, such as hypopituitarism, adrenocortical dysfunction, nephrotic syndrome, systemic lupus erythematosus, rheumatoid arthritis and other diseases need long-term use, or even lifelong use. long-term use of glucocorticoids may cause some adverse events, such as obesity, peptic ulcer, or even gastrointestinal bleeding, which can also cause high blood pressure, high plasma glucose, osteoporosis, and aseptic necrosis of the femoral head. In this study, a randomized controlled trial comparing the short-term efficacy and long-term prognosis of immediate and sequential withdrawal of methyl prednisone was performed.

The trial included more than 600 patients with COPD, and all patients were randomly assigned to either the immediate withdrawal group or the sequential withdrawal group. The general data were analyzed to ensure there were no statistically significant differences between the immediate and sequential withdrawal groups (*P* > 0.05). Follow-up results show the frequency of acute exacerbations had no statistically significant differences between the immediate and sequential withdrawal groups at 30, 90, 180, and 360 days after discharge (*P* > 0.05). The mMRC dyspnea score is a 5-point (0–4) scale based on the severity of dyspnea (Cheng et al., 2019), and the CAT score is a patient-completed instrument used to assess and quantify health-related quality of life and symptom burden in COPD patients (Jones et al., 2009). As indicators of prognosis, mMRC and CAT scores were not statistically significant difference between the immediate and sequential withdrawal groups during follow-up (*P* > 0.05). The 2011 GOLD consensus report uses symptoms, exacerbation history, and FEV1% to improve the clinical assessment and to guide treatment of COPD patients (Vestbo et al., 2012). In this study, there were no statistically significant differences in FEV1% and PEF between immediate and sequential withdrawal groups at 180 and 360 days (*P* > 0.05). The time from discharge to first acute exacerbation was significantly lower (*P* < 0.05) in the immediate withdrawal group (46.12 days) than in the sequential withdrawal group (49.02 days), indicating that sequential withdrawal of glucocorticoid can hold a longer disease stabilization time.

Different studies recommend different durations of glucocorticoid application, Yii et al. (2019) found that a history of frequent acute exacerbations is a strong and independent predictor of prognosis. In addition, the history of exacerbations is independent of severe exacerbations (Han et al., 2017). Moreover, Motegi et al. (2013) found that no index was significantly better than history of exacerbation alone to predict future exacerbations. In this study, subgroup analysis was performed according to the history of frequent acute exacerbation, but there was no statistically significant difference between the two withdrawal groups (*P* > 0.05).

The CRP is a type of acute protein that increases sharply in the plasma when the body is infected or damaged (Aksu et al., 2013). It can activate, complement, and enhance phagocytosis of phagocytes and play a regulatory role in clearing invading pathogenic microorganisms and injured, necrotic, and apoptotic cells (Sacks, 2019). The value of CRP as a prognostic biomarker in AECOPD patients has been extensively investigated (Sneh et al., 2020). The association of blood eosinophil levels with rehospitalization rates is a question that remains to be answered: some studies show increased rehospitalizations (Vedel-Krogh et al., 2016; Couillard et al., 2017), whereas others find no significant effect (Bafadhel et al., 2016). In this study, subgroup analysis was performed according to CRP and eosinophils, but there was no statistically significant difference between the two methods of withdrawal (*P* > 0.05). This result might be due to CRP having a half-life of 19 h and therefore there is no significant difference in a long-term follow-up. Thus, the study was expanded to identify the relevant indicators. The GOLD was developed to spread awareness of COPD as a major public health problem and facilitate its prevention and treatment (Nowak et al., 2020). To explore the effect of the GOLD on patient prognosis, patients were divided into high or low GOLD grade groups, and the difference was significant (*P* < 0.05). The GOLD attempts to promote spirometry not only as a case-finding and prognostication tool for airflow obstruction but also as a marker for good health (Bhatt et al., 2019). According to this study, patients with an elevated GOLD grade should be treated with sequential withdrawal.

The FeNO measurement was considered to be a biomarker for airway eosinophilic inflammation (Zhou et al., 2020), because its concentration is highly correlated with the number of inflammatory cells (Hogman et al., 2019). In this study, subgroup analysis was performed according to FeNO, and the difference was not statistically significant (*P* > 0.05). FeNO is significantly elevated in asthmatic patients (Heaney et al., 2019), and FeNO values in stable COPD patients were slightly higher than those in healthy individuals (Matsunaga et al., 2020). The relationship between FeNO and AECOPD needs to be explored further. The elderly are the main patients with chronic diseases (Zhou et al., 2019), COPD is one of the major chronic diseases. In this study, subgroup analysis was performed according to age, and the difference was significant (*P* < 0.05). The ROC curve analysis showed sequential withdrawal at age > 59 years could decrease acute exacerbation frequency at the 30-day follow-up. Elderly patients are likely to have many complications that affect treatment (Rydberg et al., 2020). Clinicians should consider increasing age as a key risk factor in the management of COPD and develop correct treatment to improve the therapeutic effect (Stone et al., 2012). Because there was no consensus for the time and dose of systemic glucocorticoids in treating AECOPD, they should be explored in further studies.

The study had some deficiencies: (1) although the study was a randomized, double-blind, controlled trial, the open labeling might have affected clinical decisions and outcome endpoints; (2) the number of patients were insufficient to distinguish causes of death, and mortality assessment was not targeted, therefore, adverse reactions of glucocorticoid therapy might not have been detected.

## Conclusion

In this study, the glucocorticoid schemes for AECOPD treatment were analyzed. sequential withdrawal of glucocorticoid can hold a longer disease stabilization time. The frequency of acute exacerbations at the 30-day follow-up was significantly higher in patients with age > 63.5 years or GOLD > 3 in the immediate withdrawal group than in the sequential withdrawal group, suggesting that the short-term efficacy was poor. AECOPD patients who are elderly or with a high GOLD grade should be treated with glucocorticoid sequential withdrawal to reduce the acute exacerbation frequency of the 30-day follow-up.

## Data Availability Statement

The original contributions presented in the study are included in the article/supplementary material, further inquiries can be directed to the corresponding author/s.

## Ethics Statement

The studies involving human participants were reviewed and approved by the Huazhong University of Science and Technology Institutional Review Board. The patients/participants provided their written informed consent to participate in this study.

## Author Contributions

All authors contributed to data analysis, drafting or revising the article, gave final approval of the version to be published, and agreed to be accountable for all aspects of the work.

## Conflict of Interest

The authors declare that the research was conducted in the absence of any commercial or financial relationships that could be construed as a potential conflict of interest.
